# Digital Alternative Communication for Individuals with Amyotrophic Lateral Sclerosis: What We Have

**DOI:** 10.3390/jcm12165235

**Published:** 2023-08-11

**Authors:** Felipe Fernandes, Ingridy Barbalho, Arnaldo Bispo Júnior, Luca Alves, Danilo Nagem, Hertz Lins, Ernano Arrais Júnior, Karilany D. Coutinho, Antônio H. F. Morais, João Paulo Q. Santos, Guilherme Medeiros Machado, Jorge Henriques, César Teixeira, Mário E. T. Dourado Júnior, Ana R. R. Lindquist, Ricardo A. M. Valentim

**Affiliations:** 1 Laboratory of Technological Innovation in Health (LAIS), Federal University of Rio Grande do Norte (UFRN), Natal 59010-090, Brazil; ingridy.marina@lais.huol.ufrn.br (I.B.); arnaldo.bispo@lais.huol.ufrn.br (A.B.J.); luca.pareja@lais.huol.ufrn.br (L.A.); danilo.nagem@lais.huol.ufrn.br (D.N.); hertz.lins@lais.huol.ufrn.br (H.L.); ernano.arrais@lais.huol.ufrn.br (E.A.J.); karilany@lais.huol.ufrn.br (K.D.C.); medourado03@gmail.com (M.E.T.D.J.); raquel.lindquist@ufrn.br (A.R.R.L.); ricardo.valentim@lais.huol.ufrn.br (R.A.M.V.); 2 Advanced Nucleus of Technological Innovation (NAVI), Federal Institute of Rio Grande do Norte (IFRN), Natal 59015-000, Brazil; higor.morais@lais.huol.ufrn.br (A.H.F.M.); joao.queiroz@navi.ifrn.edu.br (J.P.Q.S.); 3 Research Department, ECE-Engineering School, 75015 Paris, France; gui.medeiros1@gmail.com; 4 Department of Informatics Engineering, Center for Informatics and Systems of the University of Coimbra, Universidade de Coimbra, 3030-788 Coimbra, Portugal; jh@dei.uc.pt (J.H.); cteixei@dei.uc.pt (C.T.); 5 Department of Integrated Medicine, Federal University of Rio Grande do Norte (UFRN), Natal 59010-090, Brazil

**Keywords:** machine learning, computer vision, image processing, neurodegenerative diseases, chronic neurological conditions, communication assistance

## Abstract

Amyotrophic Lateral Sclerosis is a disease that compromises the motor system and the functional abilities of the person in an irreversible way, causing the progressive loss of the ability to communicate. Tools based on Augmentative and Alternative Communication are essential for promoting autonomy and improving communication, life quality, and survival. This Systematic Literature Review aimed to provide evidence on eye-image-based Human–Computer Interaction approaches for the Augmentative and Alternative Communication of people with Amyotrophic Lateral Sclerosis. The Systematic Literature Review was conducted and guided following a protocol consisting of search questions, inclusion and exclusion criteria, and quality assessment, to select primary studies published between 2010 and 2021 in six repositories: Science Direct, Web of Science, Springer, IEEE Xplore, ACM Digital Library, and PubMed. After the screening, 25 primary studies were evaluated. These studies showcased four low-cost, non-invasive Human–Computer Interaction strategies employed for Augmentative and Alternative Communication in people with Amyotrophic Lateral Sclerosis. The strategies included Eye-Gaze, which featured in 36% of the studies; Eye-Blink and Eye-Tracking, each accounting for 28% of the approaches; and the Hybrid strategy, employed in 8% of the studies. For these approaches, several computational techniques were identified. For a better understanding, a workflow containing the development phases and the respective methods used by each strategy was generated. The results indicate the possibility and feasibility of developing Human–Computer Interaction resources based on eye images for Augmentative and Alternative Communication in a control group. The absence of experimental testing in people with Amyotrophic Lateral Sclerosis reiterates the challenges related to the scalability, efficiency, and usability of these technologies for people with the disease. Although challenges still exist, the findings represent important advances in the fields of health sciences and technology, promoting a promising future with possibilities for better life quality.

## 1. Introduction

Amyotrophic Lateral Sclerosis (ALS) is a progressive and irreversible neurodegenerative disease that affects an individual’s motor neurons. As a result, there is a gradual loss of functionality in voluntary movements, respiratory function, and communication [1,2,3,4,5]. For people with ALS, having access to an ecosystem that integrates multi-professional assistance and assistive technologies, particularly Augmentative and Alternative Communication resources, has been shown to be essential to preserving communication and interaction skills and enhancing quality of life and survival as the disease advances [6,7,8,9,10,11].

As ALS progresses, functional losses intensify. Thus, the communicative process, autonomy, social interaction, and participation are partially or entirely affected. To compensate for these losses, which are related to functional and motor abilities caused by this disease, several kinds of research have been developed, with a common objective: to promote improvement in the quality of life of patients with ALS. Some of this research has been directed toward alternative communication, as this is one of the main issues for ALS patients. This is because many of these patients lose the ability to communicate, which can cause social isolation and loss of autonomy [12,13]. Therefore, research in this field is often based on Human–Computer Interaction, to promote Augmentative and Alternative Communication methods, using devices or information systems and applications within the scope of assistive technologies [14,15,16,17,18,19,20,21].

In Augmentative and Alternative Communication, there are different mechanisms and paradigms for controlling interfaces based on Human–Computer Interaction. Bioelectric signals, for instance, are widely used and investigated mechanisms in neuroscience, rehabilitation, and Brain–Computer Interface (BCI). In BCI, brain signals and electroencephalography devices are widely used for Human–Computer Interaction, especially when the person with ALS is in a locked-in state and cannot voluntarily move their eyes [22,23,24,25,26,27,28]. In a systematic review, Jaramillo-Yánez et al. [29] showed studies investigating electromyography signals for Human–Computer Interaction through muscle contractions and gesture recognition. Other studies have also suggested that, by applying electro-oculogram-based features, it is possible to control interfaces by capturing predefined eye movements (up, down, left, and right) or blinking [30,31,32,33,34,35].

Despite scientific and technological advances in the field of Augmentative and Alternative Communication using bioelectric signals, it is still a challenge to introduce devices in this category to the home environment, for use by individuals with severe motor disabilities, such as people with ALS. These limitations are related to home usability (or the ability to handle an instrument), the time necessary to select characters or items in an Augmentative and Alternative Communication interface, and the necessity of electrodes that must be attached to the patient, which usually causes fatigue and discomfort and discourages from adopting the resource [12,36,37,38].

Other approaches to Human–Computer Interaction, highlighted in significant areas of Computer Vision and Machine Learning, use computational methods based on image processing. For example, some studies have used the eyes of individuals with a severe motor disability, along with one or more cameras, to capture image data for processing and defining patterns, such as blinking [13,39,40] or pupil movement [41,42,43,44,45,46]. Thus, the result of the images’ digital processing coming from the eyes of the user acts as input for a Human–Computer Interaction system.

Fathi et al. [40] presented two categories of image-based techniques: with and without infrared. Methods for eye tracking with infrared are more effective; however, prolonged exposure to infrared may cause health damage or discomfort to the eye. Also, it may require specific hardware for the attachment to the individual’s head. The methods that use cameras without infrared lights are simpler; however, the detection aspects of eye movements or eye blinking are more complex, which may compromise the system’s accuracy, relative to the desired selection target on the interface [42,47].

In the context of Augmentative and Alternative Communication and image-based mechanisms for people with ALS or other diseases belonging to the so-called locked-in syndrome, it is challenging to craft solutions based on Human–Computer Interaction in a home environment, which requires low effort for typing and low cost as well, that are efficient and accessible. The devices for Augmentative and Alternative Communication that are considered to have high performance are usually commercial and are provided with additional elements, such as infrared and sensors for image processing, which represents high cost and robustness.

In this context, the following question arises: is it possible to build a low-cost resource, using an eye-image-based method that relies on Computer Vision and Machine Learning techniques for Augmentative and Alternative Communication, interaction, and inclusion of people with ALS? To answer this research question, this paper presents, based on the methodological features of a Systematic Literature Review [48,49,50,51,52], an investigation of primary studies that explore eye-based Human–Computer Interaction systems and technologies for Augmentative and Alternative Communication for people with ALS.

## 2. Materials and Methods

This research was developed based on the systematic review guidelines proposed by Kitchenham [48] and the Preferred Reporting Items for Systematic Reviews and Meta-Analyses (PRISMA) checklist [53], and it was registered with PROSPERO (registration no. CRD42021230721) [54]. Initially, as a fundamental part of the protocol, five Research Questions (RQ) were developed (see Table 1 below).

The process of identifying primary studies related to the investigation object of this Systematic Literature Review consisted of searches in six repositories: Science Direct, Web of Science, Springer, IEEE Xplore, ACM Digital Library, and PubMed. Searches in all databases were performed on 18 November 2021. Except for PubMed, two search strings (SS01 and SS02) were used in the searches. Specifically for PubMed, a third search string (SS03) was considered, which was defined from the Medical Subject Headings (MeSH) thesaurus. The search strings are presented below:

- SS01: (eye) AND (track OR gaze OR blink OR localization) AND (camera OR webcam) AND (“amyotrophic lateral sclerosis” OR als);
- SS02: (eye) AND (track OR gaze OR blink OR localization) AND (camera OR webcam) AND (“neuromuscular disease” OR “motor neuron disease”);
- SS03: see Appendix A.1.

After identifying and defining the initial set of records, screening was performed, to select a subset of eligible primary studies. This process was organized and executed by applying three elementary procedures: (i) Inclusion Criteria—IC; (ii) Exclusion Criteria—EC; and (iii) Quality Assessment Criteria—QA.

In procedure (i), a subset of primary studies was defined, based on the Inclusion Criteria (Table 2) applied through the filters made available in the repositories. In procedure (ii), a screening guided by the Exclusion Criteria (Table 2) based on the title reading, abstract, and keywords was performed on the subset of primary studies. Rayyan [55], a web application for systematic reviews, assisted in this step (ii).

To determine the final set of eligible articles, and to seek answers to the Research Questions (see Table 1), a screening, guided by the Quality Assessment Criteria (see Table 3), was performed from the entire reading of the primary studies. An elimination condition (QA01) and an evaluation metric, called score (see Equation (1)), were used for the qualification and ranking of the studies. The score was the arithmetic mean of the weights (*w*) assigned for each Quality Assessment Criteria. The weight (*w*), which could vary between 0, 0.5, and 1.0, measured how satisfactory the response of that article was for a given Quality Assessment Criteria, as shown in Equation (2). Primary articles that scored 0.5 or higher (i.e., 0.5≤score≤1.0) were considered eligible for this Systematic Literature Review. Two reviewers assigned scores, and elementary data from the final set of eligible studies, extracted based on the research questions, were summarized in Table 4.

(1) $$score=\frac{1}{n_{\mathrm{QA}}}\sum_{i=1}^{n_{\mathrm{QA}}}w_{{\mathrm{QA}}_{i}}$$

where:

 – $n_{QA}$: variable used to represent the total of Quality Assessment Criteria;
 – $w_{QA}$: variable used to determine the value referring to the weight *w* assigned to the Quality Assessment Criteria under analysis (see the possible values in the Equation (2)).

(2) $$w_{\mathrm{QA}}=\left\{\begin{array}{cc} 1.0, & \mathrm{yes},\mathrm{fully}\mathrm{describes},\\ 0.5, & \mathrm{yes},\mathrm{partially}\mathrm{describes},\\ 0, & \mathrm{does}\mathrm{not}\mathrm{describe}. \end{array}\right.$$

## 3. Results

**Figure 1:**
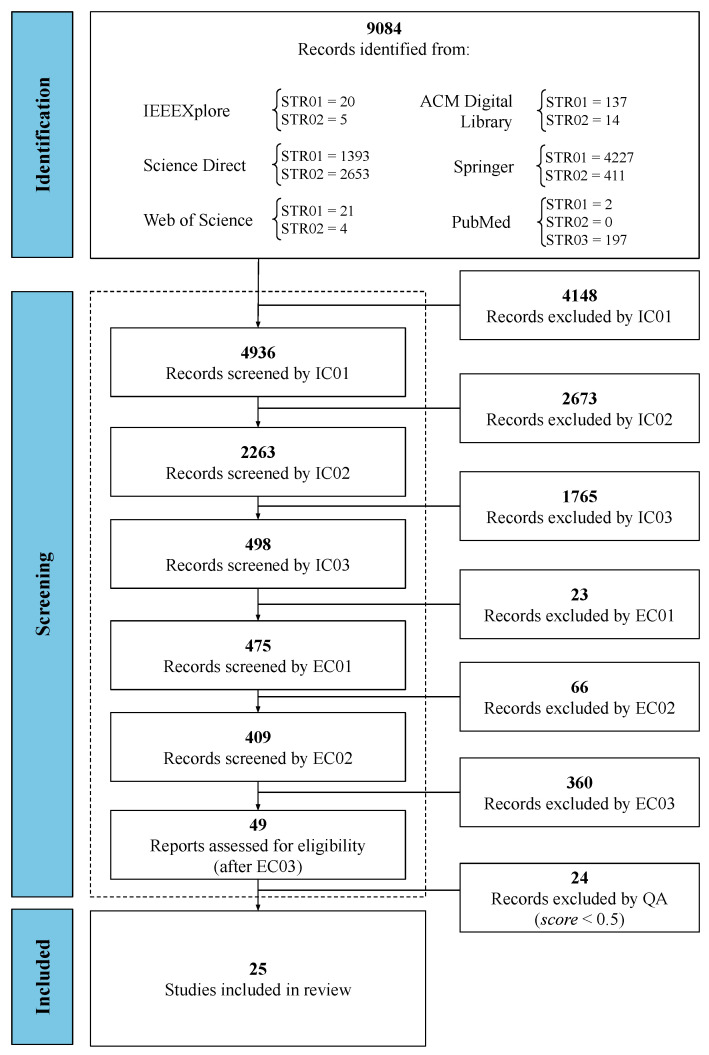
Result of the search and screening process of primary studies for this systematic review.

The detailed quantitative results of the protocol execution of this Systematic Literature Review have been summarized in Figure 1. After identifying 9084 records and performing screening, consisting of the application of Inclusion Criteria (8586 studies excluded), Exclusion Criteria (449 studies excluded), and Quality Assessment Criteria (24 studies excluded), a set of 25 primary studies were considered eligible and were included in this Systematic Literature Review, to answer the Research Questions (Table 1). The most comprehensive research results were organized and presented in the same sequence as the Research Questions. The analysis is based on the data extracted from the 25 eligible articles, briefly described in Table 4. This table was organized in groups of Human–Computer Interaction, Score, and year of publication of the articles, arranged in descending order.

### 3.1. Research Question 01

**Figure 2:**
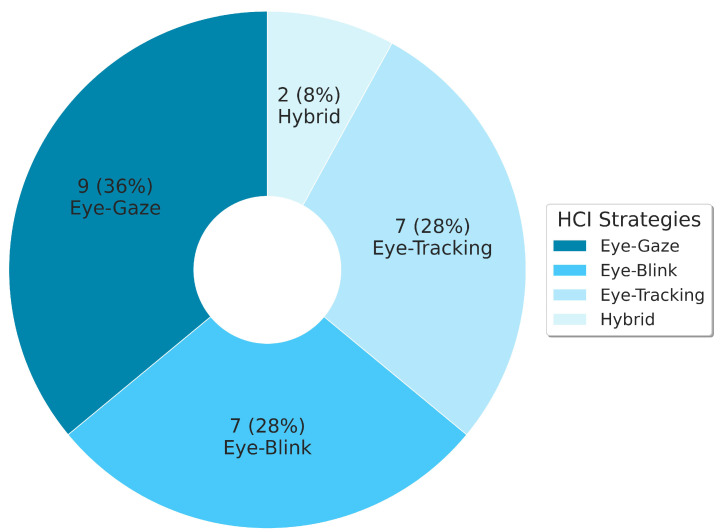
Strategies for Human–Computer Interaction (HCI) based on eye images.

Based on the primary studies, and as shown in Figure 2, the strategies evidenced for Human–Computer Interaction through the eyes are divided into four categories: Eye-Gaze; Eye-Blink; Eye-Tracking; and hybrid strategies, which combine some of the previous categories. The Human–Computer Interaction approach based on Eye-Gaze (36% of the studies) was the highlighted technique found in the research, a technique that generally seeks to estimate gaze direction based on pupil movement on the horizontal (left and right) and vertical (up and down) axes, to select the target object on the interface [56,57,58,59,60,61,62,63,64].

The Eye-Blink strategy was evidenced in 28% of the studies [65,66,67,68,69,70,71]. In this category, the approach to target selection varies and can be based on the detection/identification of voluntary eye blinking (long eye-blinks) [65], the simulation of analogous mouse clicks (right or left eye-blink) [66,67,68,70,71] and the sequential combination of blinks in a temporal space [69]. With the same percentage, 28% of the primary studies provided Human–Computer Interaction approaches to Eye-Tracking, i.e., identifying and classifying the effective pupil direction [72,73,74,75,76,77,78]. Despite its similarity to Eye-Gaze, this strategy seeks to estimate gaze direction in relation to the image pixels, which is more accurate and goes beyond horizontal and vertical lines. The studies by Zhao et al. [79] and Xu and Lin [80], which belong to the category of hybrid strategies, combine Eye-Gaze and Eye-Blink strategies.

### 3.2. Research Question 02

**Figure 3:**
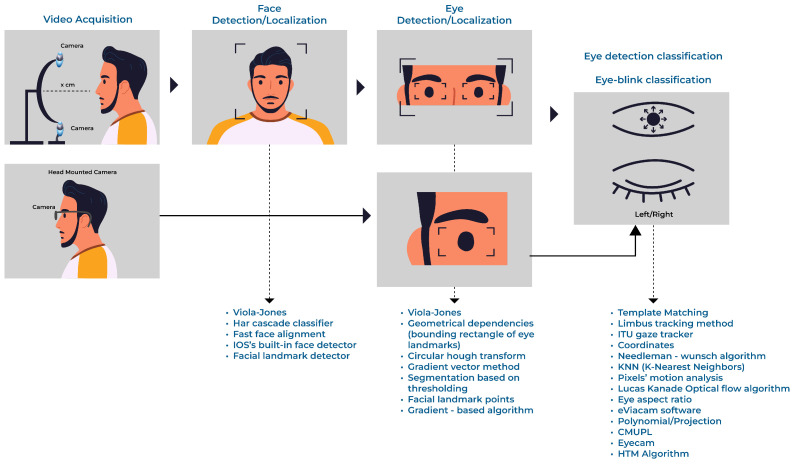
Generic workflow (pipeline) model.

The algorithmic techniques explored in the primary studies varied, due to the Human–Computer Interaction techniques presented in Section 3.1 (Research Question 01) and the environment configuration in which the camera was allocated for acquiring images/video from the user, as shown in Figure 3, in the step called Video Acquisition. Figure 3 also shows a generic workflow of the procedures (tasks) and the respective Computer Vision or Machine Learning computational techniques often used to solve the challenges comprising Human–Computer Interaction through pupil tracking or blink detection.

In the set of primary studies, mainly for the face detection/localization and eye detection/localization steps, the use of Computer Vision algorithmic resources from the Open Source Computer Vision Library (OpenCV) [81] and Dlib [82] was observed. For the face detection/localization task, for example, the authors mentioned the use of techniques based on the Viola–Jones algorithm or the Haar cascade classifier, available in the OpenCV library, and on Dlib’s built-in facial landmark detector [56,58,60,65,70,74,80]. Other sources of computational tools for image processing and Computer Vision were also explored in the face detection/localization step: for example, Singh and Singh [66] and Singh and Singh [67] used the Viola–Jones algorithm from the MathWorks company. Authors Zhang et al. [57] explored face detection with the Machine Learning Kit on iOS and landmark detection with Dlib.

The eye detection/localization task challenges were mainly related to eye clipping and pupil or iris enhancement. The computational techniques for eye clipping are intrinsically linked to the video acquisition approach, which does not have a Head-Mounted Camera and which aims to delimit or extract the region of interest (the eye). The commonly explored computational techniques were the Viola–Jones algorithm [66,67,74], algorithmic models based on Facial Landmark Points [57,70], and Geometrical Dependencies coupled with Binarization/grayscale/OpenCV strategies [56,63,65,75,78,79].

For enhancing the pupil (or iris) in an eye image, the robust and versatile technique for detecting circles called the Circular Hough Transform [58,62,63,73,74] is highlighted. Other techniques covering the eye detection/localization task can also be mentioned, such as the Limbus tracking method [59], gradient-based algorithms [60,80], Machine Learning models based on Hierarchical Temporal Memory [61,64], Erosion with a cross-shaped structure element [68], segmentation based on thresholding [69], and the Clustering Method of Unbroken Pixel Lines [76].

From the perspective of detecting eye blinking, the authors explored computational models based on Template Matching [65,68], optical flow technique and pixels’ motion analysis [66,67], mathematical formulas to calculate Eye Aspect Ratio (EAR) [70,80] or iris height and width [79], and the 2 Pixel Verification Methodology (black and white: open eye; black and black: closed eye) [69]. In addition to using the Template Matching technique, Missimer and Betke [68] incorporated the Lucas–Kanade optical flow algorithm and finite state machines into the proposed model. Krapic et al. [71] used software called eViacam with integrated implementations of Motion Analysis from the authors [83,84] and computational techniques (not specified) from the OpenCV Library.

In the set of studies where the purpose was to develop a Human–Computer Interaction strategy based on gaze direction, it is evident that the authors followed the workflow, to identify the pupil/iris in the images in advance, using varied methods such as Circular Hough Transform [58,62,63,73,74] or gradient-based algorithm [60,80], and that they extracted values related to the coordinates that served as input to mathematical models (called Geometrical Dependencies in this work) that calculated and identified the gaze direction [58,60,62,63,73,74,78,79,80]. The authors Eom et al. [56] and Yildiz et al. [62] used Geometrical Dependencies to train and create Machine Learning models, using neural networks and the K-Nearest Neighbor algorithm, respectively.

Other approaches to classify gaze direction can also be mentioned. Zhang et al. [57] used Template Matching. Abe et al. [59] explored the vertical eye-gaze detection method, which is also based on the Limbus tracking technique. Rozado et al. [61] and Rozado et al. [64] combined Machine Learning models based on Hierarchical Temporal Memory with ITU Gaze Tracker (open source library software) and the Needleman–Wunsch algorithm, respectively. In a study by Oyabu et al. [76], the pupil position was defined using the Clustering Method of Unbroken Pixel Lines. Also performing mathematical operations, Aharonson et al. [75] calculated the pupil position using two different algorithms: a parametrical interpolation-based algorithm (called a polynomial) and a model-based algorithm (called a projection). Park and Park [72] built an expert embedded system, Pupil Center Corneal Reflection, to track pupils through hardware with attached adaptive lights and a mathematical model-based program. Kaushik et al. [77] used the EyeScan software.

### 3.3. Research Question 03

The performance-related evaluation of the computational techniques explored in the primary studies showed promising results in control group testing. The analysis of the 13 studies that reported the performance of the techniques in percentage terms shows that the average accuracy (Acc) reached the value of 94.12% (std = 4.14; median = 95%) [57,58,61,63,64,65,66,67,68,74,77,79,80]. The approach proposed by authors Park and Park [72] obtained accuracy of 1–2${}^{{}^{\circ}}$. In addition to the accuracy of 95.17%, Królak and Strumiłło [65] measured Recall and Precision, which obtained values of 96.91% and 98.13%, respectively. Singh and Singh [67] also measured performance on more than one metric, showing 91.2% Acc and 94.11% Precision. Abe et al. [59] presented the average error in two perspectives of eye-gaze detection: vertical detection (0.56${}^{{}^{\circ}}$) and horizontal detection (1.09${}^{{}^{\circ}}$). The authors Rahnama-ye-Moqaddam and Vahdat-Nejad [60] reported an average error rate of 5.68% and, now looking at the best error rate obtained by the system, Yildiz et al. [62] presented 0.98% as a result. All the computational techniques explored are identified in Table 4.

Other approaches to evaluating technology performance for Human–Computer Interaction have been used. Eom et al. [56] conducted computer experiments with a control group and summed the individual participants’ gaze movement error (vertical and horizontal). Zhang et al. [57] evaluated, in addition to the Eye-Gaze system, the usability of Augmentative and Alternative Communication software, through a questionnaire, with questions based on the Likert scale [85]. Similarly, Krapic et al. [71] evaluated usability tests. Rupanagudi et al. [69] evaluated and compared the speed of the proposed algorithm to another approach in the literature. With an evaluation system based on pattern recognition, Rakshita [70] reported the efficiency of the approach (without quantifying). Saleh and Tarek [73] evaluated the proposal based on an interface with six targets representing user needs. Aharonson et al. [75] constructed a table containing each user’s mean deviation in degrees. The experimental results in Oyabu et al. [76] were presented by calculating the time, using a “click experiment screenshot” system. Kavale et al. [78] showed the performance of the techniques used through images.

### 3.4. Research Question 04

Based on the Video Acquisition step presented in Figure 3, 76% of the primary studies [56,57,58,59,60,61,65,66,67,68,69,70,71,72,74,76,78,79,80] performed computational experiments using devices for image collection placed on a table or integrated into the computer itself, as in the case of notebooks or smartphones with integrated cameras, which characterizes a Human–Computer Interaction approach where users are free of devices on their body. Alternatively, 24% of the studies explored a Human–Computer Interaction approach where the prototype for image collection, the camera, was mounted on the user’s head [62,63,64,73,75,77].

From a general perspective, 52% of the primary studies proposed Human–Computer Interaction devices equipped with some light source projected onto the user’s eye or face, being infrared lights [61,63,64,69,72,73,76,77,78,79,80] and lamps [66,67]. In the category of Human–Computer Interaction strategies based on Eye-Gaze, which accumulated the most significant number of studies (nine), five proposed Augmentative and Alternative Communication approaches (approximately 55.6%) using cameras free of additional or body features [56,57,58,59,60]. Of the other four studies in the same category, three explored Head-Mounted Camera approaches [62,63,64], with two proposing infrared [63,64], while authors Rozado et al. [61] added infrared lights to the camera.

In the Eye-Blink category, all seven studies explored image capture using cameras not mounted on the user’s head [65,66,67,68,69,70,71]. Three studies equipped the cameras with some type of light source projected onto the user’s eye or face, with one being infrared lights [69] and two being lamps [66,67]. Video acquisition in studies belonging to the Eye-Tracking category varied between approaches with Head-Mounted Cameras [73,75,77], two of them with infrared [73,77], and with non-head-mounted cameras equipped with [72,76,78] and without [74] infrared. The authors Zhao et al. [79] and Xu and Lin [80], from the hybrid Human–Computer Interaction strategies category, investigated Augmentative and Alternative Communication techniques from images collected from cameras with infrared.

### 3.5. Research Question 05

**Figure 4:**
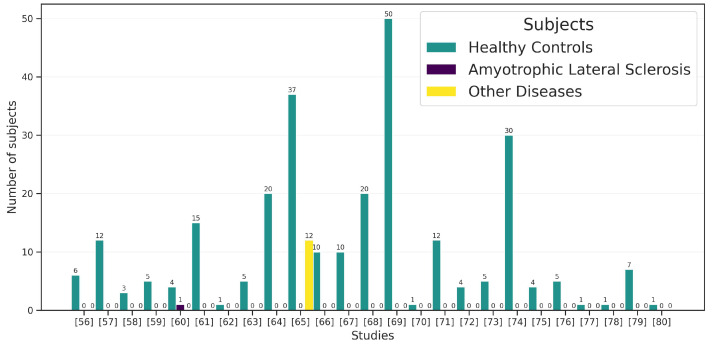
The number of subjects used in the primary studies [56,57,58,59,60,61,62,63,64,65,66,67,68,69,70,71,72,73,74,75,76,77,78,79,80].

The data extracted from the primary studies to answer these Research Questions have been summarized in Figure 4. Figure 4 clearly shows that only one study—by authors Rahnama-ye-Moqaddam and Vahdat-Nejad [60], for Eye-Gaze—performed experimental tests on a person with ALS. A second study considered participants with other (unspecified) disabilities. Królak and Strumiłło [65] included 12 people in experimental testing to evaluate the Human–Computer Interaction approach through Eye-Blink. All primary studies performed tests with healthy controls, with an average of 10.76 participants per study (std = 12.3; median = 5).

## 4. Discussion

This Systematic Literature Review investigated 25 primary studies on image-based Human–Computer Interaction approaches using simple, low-cost cameras for Augmentative and Alternative Communication for people with ALS. Initially, as an answer to the problem question, the results point to the possibility and feasibility of developing low-cost technologies for Human–Computer Interaction through eye imagery. However, there are still challenges to be explored in the broad areas of Computer Vision, Machine Learning, and Augmentative and Alternative Communication, related not only to the cost but also to the efficiency and usability of Human–Computer Interaction technologies that are used through the eyes, particularly in the context of people with ALS. From this perspective, this Systematic Literature Review organized and discussed, in sequence, the main findings.

The first analysis, related to Human–Computer Interaction strategies, showed four strategies addressed by the primary studies: Eye-Gaze (36%) [56,57,58,59,60,61,62,63,64], Eye-Blink (28%) [65,66,67,68,69,70,71], Eye-Tracking (28%) [72,73,74,75,76,77,78], and Hybrid strategies (8%) [79,80]. It was also observed that 52% of the studies adopted additional features to control the environment’s light that falls on the user’s eye or face. Of this group of studies, 11 resorted to the use of infrared [61,63,64,69,72,73,76,77,78,79,80] and 2 to fluorescent light [66,67]. The targeting of light beams at the eye aimed, in practical terms, to create reflective effects in the pupil region in the case of infrared lights, or reference points in the pupil/iris/sclera, such as to facilitate image processing and, hence, detection and classification of gaze direction or eye state (open or closed). Still, from the perspective of improving the image processing conditions, gaze motion detection, and performance of the Human–Computer Interaction device, six studies (24%) conducted experiments with the camera attached to the users’ heads [62,63,64,73,75,77].

The surveys highlighted in this study could be relevant if they aligned with sharing knowledge and the Augmentative and Alternative Communication resource (the final product). Therefore, the goal must be to improve the functional capacities of people with motor disabilities—that is, the autonomy of those individuals. In this way, it may be possible to mitigate the effects of social isolation. Moreover, it also promotes the exercise of rights, citizenship, fundamental freedoms, and health care for people with ALS. This aspect is very significant, for it acts directly on health promotion, well-being, and the reduction of inequalities, which may reflect in the promotion of equity. These factors are even foreseen in the Sustainable Development Goals (SDGs) that are part of the United Nations (UN) 2030 Agenda, particularly SDGs 3 (3.8) and 10 [86]. Therefore, this issue is not only about developing new technologies or simply providing low-cost solutions but is also about acting through technological mediation as research that induces social inclusion, reduces inequalities, and promotes equity—values often not measurable in scientific research of a more technological nature.

Although most of the results occurred in healthy controls, they suggest the viability of investment in research in this field. However, public health policymakers must prioritize research in this area, to ensure that the poorest people diagnosed with ALS have access to assistive technologies that can improve their quality of life. It is not enough to develop new technologies: it is necessary to ensure that people with ALS have access to them, regardless of their social conditions. Securing investment in research in this field is essential, not only for providing access to people with this disease - which is fundamental - but also for ensuring the sustainability and advancement of studies in this area, which is often neglected by the industry, as the market is very limited.

ALS is considered rare, and, despite efforts to seek digital health solutions, there are still significant challenges to be tackled: these include the need for more data, studies, and evidence on disease incidence and prevalence, which are essential but scarce pieces of information in the context of global health [12,87,88,89,90,91,92,93,94,95,96,97,98,99,100,101,102,103,104,105,106,107]. There are few records or epidemiological studies in Brazil, and only two studies at national level have been mentioned in the scientific literature. In 1998, Dietrich-Neto et al. [108] conducted a national survey and reported incidence and prevalence rates of 0.4 and 1.2 per 100,000 inhabitants, respectively. More recently, when analyzing the period from 2004 to 2013, the researchers Moura et al. [109] estimated the average incidence of ALS to be 0.461 cases per 100,000 inhabitants (with a trend of increasing incidence over the years), a rate similar to that of Dietrich-Neto et al. [108]. It is worth mentioning that, in Brazil, until 2019, there was no compulsory notification system or national registry of Amyotrophic Lateral Sclerosis, which may have led to under-reporting [107,110].

To address this problem of under-reporting in Brazil, Barbalho et al. [111] emphasize the National ALS Registry, an applied research project supported by the Brazilian Ministry of Health. According to Barbalho et al. [111], this National Registry is a project still in progress and under implementation throughout Brazil, the goal of which is to continuously map all people with ALS in the country online. Through the National Registry, it will be possible to develop epidemiological studies and analyses that can support the decision making of public authorities in the design of health policies in the context of ALS in Brazil, for example. In this sense, Law Project No. 4691 of 2019 [112] aimed to make the notification of rare diseases mandatory in Brazil, and so the National ALS Registry is a structuring part of this Law Project. Noteworthy in Brazil is the state of Rio Grande do Norte, which is in the Northeast Region of the country, as it was the first Brazilian state to publish Law No. 10,924 of 10 June 2021 [113], which made the notification of ALS compulsory.

There are many challenges in the ALS context, and it is clear that they go beyond the areas of health sciences and technology. However, understanding transdisciplinarity and the appropriate use of these technologies or digital health solutions could significantly improve access to quality health care, reduce inequalities, and improve life quality, especially for people with ALS. Therefore, it is also necessary to consider technologies as tools for society’s social and sustainable development.

## 5. Conclusions

This paper, through the execution of a Systematic Literature Review protocol, investigated primary studies in the literature and highlighted five relevant points that could directly contribute to development and technological effectiveness in providing eye-image-based Human–Computer Interaction strategies regarding Augmentative and Alternative Communication for people with ALS. The first point showed the Human–Computer Interaction approaches based on eye images: Eye-Gaze (36%) [56,57,58,59,60,61,62,63,64]; Eye-Blink (28%) [65,66,67,68,69,70,71]; Eye-Tracking (28%) [72,73,74,75,76,77,78]; and hybrid strategies (8%) [79,80]. These Human–Computer Interaction approaches are the results of efforts by the scientific community to develop low-cost solutions and to indicate the feasibility of their use as assistive technologies for Augmentative and Alternative Communication for people with ALS or other diseases that compromise functional abilities. The computational resources related to Computer Vision/Machine Learning techniques and to hardware support for image acquisition and enhancement were also examined and described in Table 4, which summarizes the answers to these and the other investigated points.

The computational models identified showed potential for face and eye detection and for eye movement tracking tasks or eye state classification (open or closed). However, there were limitations regarding experiments on people with ALS and, in some studies, the methodological density of the model structure and application. In addition to these limitations, it is important to highlight that computational techniques have reached an efficiency threshold (regarding performance), i.e., they are well-consolidated for Human–Computer Interaction through the eyes. However, it is worth noting that controlled computational experiments with a low and undiversified number of users may mask the actual results, presenting good results in the tests but without the ability to generalize the model. These aspects could be explored in further research and related to approaches without using a Head-Mounted Camera or infrared, which could direct other tests, considering people with ALS without causing discomfort.

The purpose of this Systematic Literature Review was to gather findings on eye-image-based Human–Computer Interaction approaches for the Augmentative and Alternative Communication of people with ALS. It is essentially optimistic research, regarding the innovation, development, and availability of low-cost technologies for universal access and significant improvements in the quality of life for people with ALS or other motor disabilities.

## Figures and Tables

**Table 1:** Research Questions.

RQ	Description
01	What strategy is used to establish Human–Computer Interaction based on eye images?
02	What computational technique is used for processing and classifying eye images (Computer Vision or Machine Learning, e.g.)?
03	What is the performance of the computational techniques explored (evaluated through accuracy, precision, sensitivity, specificity, error)?
04	What is the hardware support for image acquisition?
05	What is the profile of the group of individuals submitted to the experimental tests of the study (healthy controls, ALS, or other diseases)?

**Table 2:** Inclusion and Exclusion Criteria.

ID	Inclusion Criteria	Exclusion Criteria
01	Articles published between 2010 and 18 November 2021.	Duplicate articles.
02	Original and complete research articles published in Journals or Conferences.	Review articles.
03	Articles in the areas of technology, engineering, or computer science.	Articles not related to communication strategies through the eyes for Human–Computer Interaction based on generic cameras.

**Table 3:** Quality Assessment.

QA	Description	Eliminator
01	Is the research object of the study a Human–Computer Interaction approach based on eye images for people with ALS or Motor Neurone disease?	Yes
02	Does the study describe the approach to image processing?	No
03	Does the study describe the algorithmic technique’s performance (accuracy, precision, sensitivity, specificity, error)?	No
04	Does the study describe the hardware used for image acquisition?	No
05	Does the study perform experiments on control groups (healthy people), people with ALS, or other diseases?	No

**Table 4:** Summary of the main characteristics of the articles included in the systematic review.

Study	Year	Score	HCI	Hardware	Subjects	Techniques (Keywords)	Performance(%)			
					HC/ALS/OD		Acc	Recall	Precision	Error
Eom et al. [56]	2019	0.8	Eye-Gaze	Camera	6/0/0	Haar-like/binarization/grayscale/NN	A different approach			
Zhang et al. [57]	2017	0.8	Eye-Gaze	iPhone and iPad	12/0/0	Fast face alignment/GD/TM	86%	-	-	-
Aslam et al. [58]	2019	0.7	Eye-Gaze	Camera	3/0/0	Haar-like/CHT	100%	-	-	-
Abe et al. [59]	2011	0.7	Eye-Gaze	Camera	5/0/0	Limbus Tracking Method	-	-	-	0.56${}^{{}^{\circ}}$/1.09${}^{{}^{\circ}}$
Rahnama-ye-Moqaddam and Vahdat-Nejad [60]	2015	0.6	Eye-Gaze	Camera	4/1/0	Haar cascade/GVM/TM	-	-	-	5.68%
Rozado et al. [61]	2012	0.6	Eye-Gaze	Camera with IR	15/0/0	ITU Gaze Tracker/E-HTM	98%	-	-	-
Yildiz et al. [62]	2019	0.5	Eye-Gaze	HMC	1/0/0	CHT/KNN	-	-	-	0.98%
Nakazawa et al. [63]	2018	0.5	Eye-Gaze	HMC with IR	5/0/0	CHT	93.32%	-	-	-
Rozado et al. [64]	2012	0.5	Eye-Gaze	HMC with IR	20/0/0	HTM/Needleman–Wunsch	95%	-	-	-
Królak and Strumiłło [65]	2012	0.8	Eye-Blink	Camera	37/0/12	Viola–Jones/GD/TM	95.17%	96.91%	98.13%	-
Singh and Singh [66]	2019	0.7	Eye-Blink	Camera with light source	10/0/0	Viola–Jones/PMA	90%	-	-	-
Singh and Singh [67]	2018	0.7	Eye-Blink	Camera with light source	10/0/0	Viola–Jones/PMA	91.2%	-	94.11%	-
Missimer and Betke [68]	2010	0.7	Eye-Blink	Camera	20/0/0	TM/Optical flow algorithm	96.6%	-	-	-
Rupanagudi et al. [69]	2018	0.6	Eye-blink	Camera with IR	50/0/0	grayscale/SBT/2PVM	A different approach			
Rakshita [70]	2018	0.5	Eye-Blink	Camera	1/0/0	grayscale/FLD/EAR	A different approach			
Krapic et al. [71]	2015	0.5	Eye-Blink	Camera	12/0/0	eViacam software	A different approach			
Park and Park [72]	2016	0.8	Eye-Tracking	Camera with IR	4/0/0	Pupil Center Corneal Reflection	1–2${}^{{}^{\circ}}$	-	-	-
Saleh and Tarek [73]	2021	0.7	Eye-Tracking	HMC with IR	5/0/0	grayscale/CHT/GD	A different approach			
Atasoy et al. [74]	2016	0.7	Eye-Tracking	Camera	30/0/0	Viola–-Jones/grayscale/CHT/GD	90%	-	-	-
Aharonson et al. [75]	2020	0.6	Eye-Tracking	HMC	4/0/0	OpenCV/Polynomial/Projection	A different approach			
Oyabu et al. [76]	2012	0.6	Eye-Tracking	Camera with IR	5/0/0	Binarization/CMUPL	A different approach			
Kaushik et al. [77]	2018	0.5	Eye-Tracking	HMC with IR	1/0/0	EyeScan software	95%	-	-	-
Kavale et al. [78]	2018	0.5	Eye-Tracking	Camera with IR	1/0/0	Binarization/GD	A different approach			
Zhao et al. [79]	2015	0.8	Hybrid	Camera with IR	7/0/0	Binarization/GD	92.69%	-	-	-
Xu and Lin [80]	2017	0.7	Hybrid	Camera with IR	1/0/0	FLD/GD	100%	-	-	-

Abbreviations: HCI (Human–Computer Interaction), HC (healthy controls), ALS (Amyotrophic Lateral Sclerosis), OD (other diseases), Acc (accuracy), HMC (Head-Mounted Camera), IR (infrared), HTM (Hierarchical Temporal Memory), E-HTM (Extended HTM), NN (Neural Network), GVM (Gradient Vector Method), TM (Template Matching), GD (Geometrical Dependencies), CHT (Circular Hough Transform), KNN (K-Nearest Neighbor), PMA (Pixels’ Motion Analysis), SBT (Segmentation Based on Thresholding), 2PVM (2 Pixel Verification Methodology), FLD (Facial Landmark Detector), EAR (Eye Aspect Ratio), POLYNOMIAL (parametrical interpolation-based algorithm), PROJECTION (model-based algorithm), CMUPL (Clustering Method of Unbroken Pixel Lines).

## Appendix A

### Appendix A.1

Search String 03 (SS03) for the PubMed repository:

((((((Eye Tracking Technology) OR (Eye-Tracking Technologies) OR (Technology, Eye-Tracking) OR (Eyetracking Technology) OR (Eyetracking Technologies) OR (Gaze-Tracking Technology) OR (Gaze Tracking Technology) OR (Gaze-Tracking Technologies) OR (Eye-Tracking System) OR (Eye Tracking System) OR (Eye-Tracking Systems) OR (Eyetracking System) OR (Eyetracking Systems) OR (Eye Movement Data Analysis) OR (Gaze-Tracking) OR (Gaze Tracking) OR (Gaze-Tracking System) OR (Gaze Tracking System) OR (Gaze-Tracking Systems) OR (Gazetracking System) OR (Gazetracking Systems) OR (Eye-Tracking) OR (Eye Tracking)) OR ((Eye Movement) OR (Movement, Eye) OR (Movements, Eye))) OR ((Eye Movement Measurement) OR (Measurement, Eye Movement) OR (Measurements, Eye Movement))) OR ((Focusing, Ocular) OR (Ocular Focusing) OR (Ocular Fixation) OR (Eye Gaze) OR (Eye Gazes) OR (Gaze, Eye) OR (Gazes, Eye))) OR ((Saccade) OR (Saccadic Eye Movements) OR (Eye Movement, Saccadic) OR (Eye Movements, Saccadic) OR (Movement, Saccadic Eye) OR (Movements, Saccadic Eye) OR (Saccadic Eye Movement) OR (Pursuit, Saccadic) OR (Pursuits, Saccadic) OR (Saccadic Pursuit) OR (Saccadic Pursuits))) AND ((Sclerosis, Amyotrophic Lateral) OR (Gehrig’s Disease) OR (Gehrig Disease) OR (Gehrigs Disease) OR (Charcot Disease) OR (Motor Neuron Disease, Amyotrophic Lateral Sclerosis) OR (Lou Gehrig’s Disease) OR (Lou-Gehrigs Disease) OR (Disease, Lou-Gehrigs) OR (ALS - Amyotrophic Lateral Sclerosis) OR (ALS Amyotrophic Lateral Sclerosis) OR (Lou Gehrig Disease) OR (Amyotrophic Lateral Sclerosis, Guam Form) OR (Amyotrophic Lateral Sclerosis-Parkinsonism-Dementia Complex 1) OR (Amyotrophic Lateral Sclerosis Parkinsonism Dementia Complex 1) OR (Guam Form of Amyotrophic Lateral Sclerosis) OR (Guam Disease) OR (Disease, Guam) OR (Amyotrophic Lateral Sclerosis, Parkinsonism-Dementia Complex of Guam) OR (Amyotrophic Lateral Sclerosis, Parkinsonism Dementia Complex of Guam) OR (Amyotrophic Lateral Sclerosis With Dementia) OR (Dementia With Amyotrophic Lateral Sclerosis)).

## References

[1] Goutman S.A., Hardiman O., Al-Chalabi A., Chió A., Savelieff M.G., Kiernan M.C., Feldman E.L. (2022). Recent advances in the diagnosis and prognosis of amyotrophic lateral sclerosis. Lancet Neurol..

[2] Goutman S.A., Hardiman O., Al-Chalabi A., Chió A., Savelieff M.G., Kiernan M.C., Feldman E.L. (2022). Emerging insights into the complex genetics and pathophysiology of amyotrophic lateral sclerosis. Lancet Neurol..

[3] Saadeh W., Altaf M.A.B., Butt S.A. A wearable neuro-degenerative diseases detection system based on gait dynamics. Proceedings of the 2017 IFIP/IEEE International Conference on Very Large Scale Integration (VLSI-SoC).

[4] Hardiman O., Al-Chalabi A., Chio A., Corr E.M., Logroscino G., Robberecht W., Shaw P.J., Simmons Z., van den Berg L.H. (2017). Amyotrophic lateral sclerosis. Nat. Rev. Dis. Prim..

[5] van Es M.A., Hardiman O., Chio A., Al-Chalabi A., Pasterkamp R.J., Veldink J.H., van den Berg L.H. (2017). Amyotrophic lateral sclerosis. Lancet.

[6] Londral A., Pinto A., Pinto S., Azevedo L., De Carvalho M. (2015). Quality of life in amyotrophic lateral sclerosis patients and caregivers: Impact of assistive communication from early stages. Muscle Nerve.

[7] Linse K., Aust E., Joos M., Hermann A. (2018). Communication Matters—Pitfalls and Promise of Hightech Communication Devices in Palliative Care of Severely Physically Disabled Patients With Amyotrophic Lateral Sclerosis. Front. Neurol..

[8] Linse K., Rüger W., Joos M., Schmitz-Peiffer H., Storch A., Hermann A. (2018). Usability of eyetracking computer systems and impact on psychological wellbeing in patients with advanced amyotrophic lateral sclerosis. Amyotroph. Lateral Scler. Front. Degener..

[9] Rosa Silva J.P., Santiago Júnior J.B., dos Santos E.L., de Carvalho F.O., de França Costa I.M.P., de Mendonça D.M.F. (2020). Quality of life and functional independence in amyotrophic lateral sclerosis: A systematic review. Neurosci. Biobehav. Rev..

[10] Gillespie J., Przybylak-Brouillard A., Watt C.L. (2021). The Palliative Care Information Needs of Patients with Amyotrophic Lateral Sclerosis and their Informal Caregivers: A Scoping Review. J. Pain Symptom Manag..

[11] Howard I.M., Burgess K. (2021). Telehealth for Amyotrophic Lateral Sclerosis and Multiple Sclerosis. Phys. Med. Rehabil. Clin. N. Am..

[12] Fernandes F., Barbalho I., Barros D., Valentim R., Teixeira C., Henriques J., Gil P., Dourado Júnior M. (2021). Biomedical signals and machine learning in amyotrophic lateral sclerosis: A systematic review. Biomed. Eng. Online.

[13] de Lima Medeiros P.A., da Silva G.V.S., dos Santos Fernandes F.R., Sánchez-Gendriz I., Lins H.W.C., da Silva Barros D.M., Nagem D.A.P., de Medeiros Valentim R.A. (2022). Efficient machine learning approach for volunteer eye-blink detection in real-time using webcam. Expert Syst. Appl..

[14] Caligari M., Godi M., Guglielmetti S., Franchignoni F., Nardone A. (2013). Eye tracking communication devices in amyotrophic lateral sclerosis: Impact on disability and quality of life. Amyotroph. Lateral Scler. Front. Degener..

[15] Hwang C.S., Weng H.H., Wang L.F., Tsai C.H., Chang H.T. (2014). An Eye-Tracking Assistive Device Improves the Quality of Life for ALS Patients and Reduces the Caregivers’ Burden. J. Mot. Behav..

[16] Shravani T., Sai R., Vani Shree M., Amudha J., Jyotsna C., Smys S., Tavares J.M.R.S., Balas V.E., Iliyasu A.M. (2020). Assistive Communication Application for Amyotrophic Lateral Sclerosis Patients. Computational Vision and Bio-Inspired Computing.

[17] Eicher C., Kiselev J., Brukamp K., Kiemel D., Spittel S., Maier A., Oleimeulen U., Greuèl M., Antona M., Stephanidis C. (2019). Expectations and Concerns Emerging from Experiences with Assistive Technology for ALS Patients. Universal Access in Human-Computer Interaction. Theory, Methods and Tools.

[18] Sigafoos J., Schlosser R.W., Lancioni G.E., O’Reilly M.F., Green V.A., Singh N.N., Lancioni G.E., Singh N.N. (2014). Assistive Technology for People with Communication Disorders. Assistive Technologies for People with Diverse Abilities. Autism and Child Psychopathology Series.

[19] Bona S., Donvito G., Cozza F., Malberti I., Vaccari P., Lizio A., Greco L., Carraro E., Sansone V.A., Lunetta C. (2019). The development of an augmented reality device for the autonomous management of the electric bed and the electric wheelchair for patients with amyotrophic lateral sclerosis: A pilot study. Disabil. Rehabil. Assist. Technol..

[20] Santana G. A., Ortiz C O., Acosta J.F., Andaluz V.H., Kim K.J., Kim H., Baek N. (2018). Autonomous Assistance System for People with Amyotrophic Lateral Sclerosis. IT Convergence and Security 2017.

[21] Elliott M.A., Malvar H., Maassel L.L., Campbell J., Kulkarni H., Spiridonova I., Sophy N., Beavers J., Paradiso A., Needham C. (2019). Eye-controlled, power wheelchair performs well for ALS patients. Muscle Nerve.

[22] Ramakrishnan J., Mavaluru D., Sakthivel R.S., Alqahtani A.S., Mubarakali A., Retnadhas M. (2022). Brain–computer interface for amyotrophic lateral sclerosis patients using deep learning network. Neural Comput. Appl..

[23] Miao Y., Yin E., Allison B.Z., Zhang Y., Chen Y., Dong Y., Wang X., Hu D., Chchocki A., Jin J. (2020). An ERP-based BCI with peripheral stimuli: Validation with ALS patients. Cogn. Neurodynam..

[24] Sorbello R., Tramonte S., Giardina M.E., La Bella V., Spataro R., Allison B., Guger C., Chella A. (2018). A Human–Humanoid Interaction Through the Use of BCI for Locked-In ALS Patients Using Neuro-Biological Feedback Fusion. IEEE Trans. Neural Syst. Rehabil. Eng..

[25] Liu Y.H., Huang S., Huang Y.D. (2017). Motor imagery EEG classification for patients with amyotrophic lateral sclerosis using fractal dimension and Fisher’s criterion-based channel selection. Sensors.

[26] Vansteensel M.J., Pels E.G., Bleichner M.G., Branco M.P., Denison T., Freudenburg Z.V., Gosselaar P., Leinders S., Ottens T.H., Van Den Boom M.A. (2016). Fully Implanted Brain–Computer Interface in a Locked-In Patient with ALS. N. Engl. J. Med..

[27] Mainsah B.O., Collins L.M., Colwell K.A., Sellers E.W., Ryan D.B., Caves K., Throckmorton C.S. (2015). Increasing BCI communication rates with dynamic stopping towards more practical use: An ALS study. J. Neural Eng..

[28] McCane L.M., Sellers E.W., McFarland D.J., Mak J.N., Carmack C.S., Zeitlin D., Wolpaw J.R., Vaughan T.M. (2014). Brain-computer interface (BCI) evaluation in people with amyotrophic lateral sclerosis. Amyotroph. Lateral Scler. Front. Degener..

[29] Jaramillo-Yánez A., Benalcázar M.E., Mena-Maldonado E. (2020). Real-Time Hand Gesture Recognition Using Surface Electromyography and Machine Learning: A Systematic Literature Review. Sensors.

[30] Tonin A., Jaramillo-Gonzalez A., Rana A., Khalili-Ardali M., Birbaumer N., Chaudhary U. (2020). Auditory Electrooculogram-based Communication System for ALS Patients in Transition from Locked-in to Complete Locked-in State. Sci. Rep..

[31] Zhang R., He S., Yang X., Wang X., Li K., Huang Q., Yu Z., Zhang X., Tang D., Li Y. (2019). An EOG-Based Human-Machine Interface to Control a Smart Home Environment for Patients with Severe Spinal Cord Injuries. IEEE Trans. Biomed. Eng..

[32] Chang W.D., Cha H.S., Kim D.Y., Kim S.H., Im C.H. (2017). Development of an electrooculogram-based eye-computer interface for communication of individuals with amyotrophic lateral sclerosis. J. Neuroeng. Rehabil..

[33] Larson A., Herrera J., George K., Matthews A. Electrooculography based electronic communication device for individuals with ALS. Proceedings of the 2017 IEEE Sensors Applications Symposium (SAS).

[34] Lingegowda D.R., Amrutesh K., Ramanujam S. Electrooculography based assistive technology for ALS patients. Proceedings of the 2017 IEEE International Conference on Consumer Electronics-Asia (ICCE-Asia).

[35] Pinheiro C.G., Naves E.L., Pino P., Losson E., Andrade A.O., Bourhis G. (2011). Alternative communication systems for people with severe motor disabilities: A survey. Biomed. Eng. Online.

[36] Chaudhary U., Vlachos I., Zimmermann J.B., Espinosa A., Tonin A., Jaramillo-Gonzalez A., Khalili-Ardali M., Topka H., Lehmberg J., Friehs G.M. (2022). Spelling interface using intracortical signals in a completely locked-in patient enabled via auditory neurofeedback training. Nat. Commun..

[37] Singh H., Singh J. (2019). Object Acquisition and Selection in Human Computer Interaction Systems: A Review. Int. J. Intell. Syst. Appl. Eng..

[38] Chaudhary U., Birbaumer N., Ramos-Murguialday A. (2016). Brain—Computer interfaces for communication and rehabilitation. Nat. Rev. Neurol..

[39] Kayadibi I., Güraksın G.E., Ergün U., Özmen Süzme N. (2022). An Eye State Recognition System Using Transfer Learning: AlexNet-Based Deep Convolutional Neural Network. Int. J. Comput. Intell. Syst..

[40] Fathi A., Abdali-Mohammadi F. (2015). Camera-based eye blinks pattern detection for intelligent mouse. Signal Image Video Process..

[41] Mu S., Shibata S., Chun Chiu K., Yamamoto T., kuan Liu T. (2022). Study on eye-gaze input interface based on deep learning using images obtained by multiple cameras. Comput. Electr. Eng..

[42] Hwang I.S., Tsai Y.Y., Zeng B.H., Lin C.M., Shiue H.S., Chang G.C. (2021). Integration of eye tracking and lip motion for hands-free computer access. Univers. Access Inf. Soc..

[43] Blignaut P. (2017). Development of a gaze-controlled support system for a person in an advanced stage of multiple sclerosis: A case study. Univers. Access Inf. Soc..

[44] Chareonsuk W., Kanhaun S., Khawkam K., Wongsawang D. Face and Eyes mouse for ALS Patients. Proceedings of the 2016 Fifth ICT International Student Project Conference (ICT-ISPC).

[45] Liu S.S., Rawicz A., Ma T., Zhang C., Lin K., Rezaei S., Wu E. (2012). An Eye-Gaze Tracking and Human Computer Interface System for People with ALS and Other Locked-in Diseases. J. Med. Biol. Eng..

[46] Liu Y., Lee B.S., Rajan D., Sluzek A., McKeown M.J. (2019). CamType: Assistive text entry using gaze with an off-the-shelf webcam. Mach. Vis. Appl..

[47] Holmqvist K., Örbom S.L., Hooge I.T.C., Niehorster D.C., Alexander R.G., Andersson R., Benjamins J.S., Blignaut P., Brouwer A.M., Chuang L.L. (2023). Eye tracking: Empirical foundations for a minimal reporting guideline. Behav. Res. Methods.

[48] Kitchenham B. (2004). Procedures for Performing Systematic Reviews.

[49] Brereton P., Kitchenham B.A., Budgen D., Turner M., Khalil M. (2007). Lessons from applying the systematic literature review process within the software engineering domain. J. Syst. Softw..

[50] Kitchenham B.A., Budgen D., Brereton P. (2016). Evidence-Based Software Engineering and Systematic Reviews.

[51] Snyder H. (2019). Literature review as a research methodology: An overview and guidelines. J. Bus. Res..

[52] Keele S. (2007). Guidelines for Performing Systematic Literature Reviews in Software Engineering.

[53] Page M.J., McKenzie J.E., Bossuyt P.M., Boutron I., Hoffmann T.C., Mulrow C.D., Shamseer L., Tetzlaff J.M., Akl E.A., Brennan S.E. (2021). The PRISMA 2020 statement: An updated guideline for reporting systematic reviews. BMJ.

[54] Fernandes F., Barbalho I. Camera-Based Eye Interaction Techniques for Amyotrophic Lateral Sclerosis Individuals: A Systematic Review. PROSPERO. 2021. CRD42021230721. https://www.crd.york.ac.uk/prospero/display_record.php?RecordID=230721.

[55] Ouzzani M., Hammady H., Fedorowicz Z., Elmagarmid A. (2016). Rayyan—A web and mobile app for systematic reviews. Syst. Rev..

[56] Eom Y., Mu S., Satoru S., Liu T. A Method to Estimate Eye Gaze Direction When Wearing Glasses. Proceedings of the 2019 International Conference on Technologies and Applications of Artificial Intelligence (TAAI).

[57] Zhang X., Kulkarni H., Morris M.R. Smartphone-Based Gaze Gesture Communication for People with Motor Disabilities. Proceedings of the 2017 CHI Conference on Human Factors in Computing Systems, CHI ’17.

[58] Aslam Z., Junejo A.Z., Memon A., Raza A., Aslam J., Thebo L.A. Optical Assistance for Motor Neuron Disease (MND) Patients Using Real-time Eye Tracking. Proceedings of the 2019 8th International Conference on Information and Communication Technologies (ICICT).

[59] Abe K., Ohi S., Ohyama M., Jacko J.A. (2011). Eye-gaze Detection by Image Analysis under Natural Light. Human-Computer Interaction. Interaction Techniques and Environments.

[60] Rahnama-ye Moqaddam R., Vahdat-Nejad H. Designing a pervasive eye movement-based system for ALS and paralyzed patients. Proceedings of the 2015 5th International Conference on Computer and Knowledge Engineering (ICCKE).

[61] Rozado D., Rodriguez F.B., Varona P. (2012). Low cost remote gaze gesture recognition in real time. Appl. Soft Comput..

[62] Yildiz M., Yorulmaz M. Gaze-Controlled Turkish Virtual Keyboard Application with Webcam. Proceedings of the 2019 Medical Technologies Congress (TIPTEKNO).

[63] Nakazawa N., Aikawa S., Matsui T. Development of Communication Aid Device for Disabled Persons Using Corneal Surface Reflection Image. Proceedings of the 2nd International Conference on Graphics and Signal Processing, ICGSP’18.

[64] Rozado D., Agustin J.S., Rodriguez F.B., Varona P. (2012). Gliding and Saccadic Gaze Gesture Recognition in Real Time. ACM Trans. Interact. Intell. Syst..

[65] Królak A., Strumiłło P. (2012). Eye-blink detection system for human–computer interaction. Univers. Access Inf. Soc..

[66] Singh H., Singh J. (2019). Object acquisition and selection using automatic scanning and eye blinks in an HCI system. J. Multimodal User Interfaces.

[67] Singh H., Singh J. (2018). Real-time eye blink and wink detection for object selection in HCI systems. J. Multimodal User Interfaces.

[68] Missimer E., Betke M. Blink and Wink Detection for Mouse Pointer Control. Proceedings of the 3rd International Conference on PErvasive Technologies Related to Assistive Environments, PETRA ’10.

[69] Rupanagudi S.R., Bhat V.G., Ranjani B.S., Srisai A., Gurikar S.K., Pranay M.R., Chandana S. A simplified approach to assist motor neuron disease patients to communicate through video oculography. Proceedings of the 2018 International Conference on Communication information and Computing Technology (ICCICT).

[70] Rakshita R. Communication Through Real-Time Video Oculography Using Face Landmark Detection. Proceedings of the 2018 Second International Conference on Inventive Communication and Computational Technologies (ICICCT).

[71] Krapic L., Lenac K., Ljubic S. (2015). Integrating Blink Click interaction into a head tracking system: Implementation and usability issues. Univers. Access Inf. Soc..

[72] Park J.H., Park J.B. (2016). A novel approach to the low cost real time eye mouse. Comput. Stand. Interfaces.

[73] Saleh N., Tarek A. Vision-Based Communication System for Patients with Amyotrophic Lateral Sclerosis. Proceedings of the 2021 3rd Novel Intelligent and Leading Emerging Sciences Conference (NILES).

[74] Atasoy N.A., Çavuşoğlu A., Atasoy F. (2016). Real-Time motorized electrical hospital bed control with eye-gaze tracking. Turk. J. Electr. Eng. Comput. Sci..

[75] Aharonson V., Coopoo V.Y., Govender K.L., Postema M. (2020). Automatic pupil detection and gaze estimation using the vestibulo-ocular reflex in a low-cost eye-tracking setup. SAIEE Afr. Res. J..

[76] Oyabu Y., Takano H., Nakamura K. Development of the eye input device using eye movement obtained by measuring the center position of the pupil. Proceedings of the 2012 IEEE International Conference on Systems, Man, and Cybernetics (SMC).

[77] Kaushik R., Arora T., Tripathi R. Design of Eyewriter for ALS Patients throughEyecan. Proceedings of the 2018 International Conference on Advances in Computing, Communication Control and Networking (ICACCCN).

[78] Kavale K., Kokambe K., Jadhav S. taskEYE: “A Novel Approach to Help People Interact with Their Surrounding Through Their Eyes”. Proceedings of the 2018 IEEE 18th International Conference on Advanced Learning Technologies (ICALT).

[79] Zhao Q., Yuan X., Tu D., Lu J. (2015). Eye moving behaviors identification for gaze tracking interaction. J. Multimodal User Interfaces.

[80] Xu C.L., Lin C.Y. Eye-motion detection system for mnd patients. Proceedings of the 2017 IEEE 4th International Conference on Soft Computing & Machine Intelligence (ISCMI).

[81] Bradski G.R. (1998). Computer vision face tracking for use in a perceptual user interface. Intel Technol. J..

[82] King D.E. (2009). Dlib-ml: A Machine Learning Toolkit. J. Mach. Learn. Res..

[83] Grauman K., Betke M., Gips J., Bradski G. Communication via eye blinks - detection and duration analysis in real time. Proceedings of the 2001 IEEE Computer Society Conference on Computer Vision and Pattern Recognition, CVPR 2001.

[84] Chau M., Betke M. Real Time Eye Tracking and Blink Detection with USB Cameras. Technical Report, Boston University. CAS: Computer Science: Technical Reports. OpenBU: Boston, MA, USA, 2005. OpenBU. https://open.bu.edu/handle/2144/1839.

[85] Likert R. (1932). A technique for the measurement of attitudes. Arch. Psychol..

[86] United Nations Take Action for the Sustainable Development Goals. New York, NY, USA..

[87] Papaiz F., Dourado M.E.T., Valentim R.A.d.M., de Morais A.H.F., Arrais J.P. (2022). Machine Learning Solutions Applied to Amyotrophic Lateral Sclerosis Prognosis: A Review. Front. Comput. Sci..

[88] Gromicho M., Leão T., Oliveira Santos M., Pinto S., Carvalho A.M., Madeira S.C., De Carvalho M. (2022). Dynamic Bayesian networks for stratification of disease progression in amyotrophic lateral sclerosis. Eur. J. Neurol..

[89] Tavazzi E., Daberdaku S., Zandonà A., Vasta R., Nefussy B., Lunetta C., Mora G., Mandrioli J., Grisan E., Tarlarini C. (2022). Predicting functional impairment trajectories in amyotrophic lateral sclerosis: A probabilistic, multifactorial model of disease progression. J. Neurol..

[90] Gordon J., Lerner B. (2019). Insights into Amyotrophic Lateral Sclerosis from a Machine Learning Perspective. J. Clin. Med..

[91] Ahangaran M., Chiò A., Lidströmer N., Ashrafian H. (2020). AIM in Amyotrophic Lateral Sclerosis. Artificial Intelligence in Medicine.

[92] Dalgıç Ö.O., Wu H., Safa Erenay F., Sir M.Y., Özaltın O.Y., Crum B.A., Pasupathy K.S. (2021). Mapping of critical events in disease progression through binary classification: Application to amyotrophic lateral sclerosis. J. Biomed. Inform..

[93] Ahangaran M., Chiò A., D’Ovidio F., Manera U., Vasta R., Canosa A., Moglia C., Calvo A., Minaei-Bidgoli B., Jahed-Motlagh M.R. (2022). Causal associations of genetic factors with clinical progression in amyotrophic lateral sclerosis. Comput. Methods Programs Biomed..

[94] Bede P., Murad A., Hardiman O. (2022). Pathological neural networks and artificial neural networks in ALS: Diagnostic classification based on pathognomonic neuroimaging features. J. Neurol..

[95] Iadanza E., Fabbri R., Goretti F., Nardo G., Niccolai E., Bendotti C., Amedei A. (2022). Machine learning for analysis of gene expression data in fast- and slow-progressing amyotrophic lateral sclerosis murine models. Biocybern. Biomed. Eng..

[96] Thome J., Steinbach R., Grosskreutz J., Durstewitz D., Koppe G. (2022). Classification of amyotrophic lateral sclerosis by brain volume, connectivity, and network dynamics. Hum. Brain Mapp..

[97] Greco A., Chiesa M.R., Da Prato I., Romanelli A.M., Dolciotti C., Cavallini G., Masciandaro S.M., Scilingo E.P., Del Carratore R., Bongioanni P. (2021). Using blood data for the differential diagnosis and prognosis of motor neuron diseases: A new dataset for machine learning applications. Sci. Rep..

[98] Kocar T.D., Behler A., Ludolph A.C., Müller H.P., Kassubek J. (2021). Multiparametric Microstructural MRI and Machine Learning Classification Yields High Diagnostic Accuracy in Amyotrophic Lateral Sclerosis: Proof of Concept. Front. Neurol..

[99] Leão T., Madeira S.C., Gromicho M., de Carvalho M., Carvalho A.M. (2021). Learning dynamic Bayesian networks from time-dependent and time-independent data: Unraveling disease progression in Amyotrophic Lateral Sclerosis. J. Biomed. Inform..

[100] Grollemund V., Le Chat G., Secchi-Buhour M.S., Delbot F., Pradat-Peyre J.F., Bede P., Pradat P.F. (2021). Manifold learning for amyotrophic lateral sclerosis functional loss assessment. J. Neurol..

[101] Kocar T.D., Müller H.P., Ludolph A.C., Kassubek J. (2021). Feature selection from magnetic resonance imaging data in ALS: A systematic review. Ther. Adv. Chronic Dis..

[102] Grollemund V., Chat G.L., Secchi-Buhour M.S., Delbot F., Pradat-Peyre J.F., Bede P., Pradat P.F. (2020). Development and validation of a 1-year survival prognosis estimation model for Amyotrophic Lateral Sclerosis using manifold learning algorithm UMAP. Sci. Rep..

[103] Myszczynska M.A., Ojamies P.N., Lacoste A.M.B., Neil D., Saffari A., Mead R., Hautbergue G.M., Holbrook J.D., Ferraiuolo L. (2020). Applications of machine learning to diagnosis and treatment of neurodegenerative diseases. Nat. Rev. Neurol..

[104] Chen Q.F., Zhang X.H., Huang N.X., Chen H.J. (2020). Identification of Amyotrophic Lateral Sclerosis Based on Diffusion Tensor Imaging and Support Vector Machine. Front. Neurol..

[105] Grollemund V., Pradat P.F., Querin G., Delbot F., Le Chat G., Pradat-Peyre J.F., Bede P. (2019). Machine Learning in Amyotrophic Lateral Sclerosis: Achievements, Pitfalls, and Future Directions. Front. Neurosci..

[106] Pinto S., Quintarelli S., Silani V. (2020). New technologies and Amyotrophic Lateral Sclerosis – Which step forward rushed by the COVID-19 pandemic?. J. Neurol. Sci..

[107] Barbalho I., Valentim R., Júnior M.D., Barros D., Júnior H.P., Fernandes F., Teixeira C., Lima T., Paiva J., Nagem D. (2021). National registry for amyotrophic lateral sclerosis: A systematic review for structuring population registries of motor neuron diseases. BMC Neurol..

[108] Dietrich-Neto F., Callegaro D., Dias-Tosta E., Silva H.A., Ferraz M.E., Lima J.M.B.D., Oliveira A.S.B. (2000). Amyotrophic lateral sclerosis in Brazil: 1998 national survey. Arq. Neuro-Psiquiatr..

[109] Moura M.C., Casulari L.A., Novaes M.R.C.G. (2016). Ethnic and demographic incidence of amyotrophic lateral sclerosis (ALS) in Brazil: A population based study. Amyotroph. Lateral Scler. Front. Degener..

[110] Barbalho I.M.P., Fernandes F., Barros D.M.S., Paiva J.C., Henriques J., Morais A.H.F., Coutinho K.D., Coelho Neto G.C., Chioro A., Valentim R.A.M. (2022). Electronic health records in Brazil: Prospects and technological challenges. Front. Public Health.

[111] Barbalho I.M.P., Fonseca A., Fernandes F., Henriques J., Gil P., Nagem D., Lindquist R., Santos-Lima T., Santos J.P.Q., Paiva J.C. (2023). Digital Health Solution for Monitoring and Surveillance of Amyotrophic Lateral Sclerosis in Brazil. Front. Public Health..

[112] Brasil Projeto de Lei N° 4691, De 2019. Atividade Legislativa. Senado Federal. Brasília, DF. https://www25.senado.leg.br/web/atividade/materias/-/materia/138326.

[113] Brasil Lei N° 10924, De 10 de Junho de 2021. Diário Oficial do Rio Grande do Norte. http://diariooficial.rn.gov.br/dei/dorn3/docview.aspx?id_jor=00000001&data=20210611&id_doc=726286..

